# Numerical Investigation of Damage Evolution in SiC/Al Composites Under Quasi-Static Tension Using the GTN Model

**DOI:** 10.3390/ma19143103

**Published:** 2026-07-19

**Authors:** Jingquan Li, Guoqiu He, Xiaoshan Liu, Yiping Liao, Yinfu Liu

**Affiliations:** 1 School of Materials Science and Engineering, Tongji University, Shanghai 201804, China; 2 Shanghai Key Laboratory for Research and Development and Application of Metallic Functional Materials, Shanghai 201804, China

**Keywords:** aluminum matrix composites, GTN model, ductile damage, strain rate, finite element analysis

## Abstract

This study employed the Gurson–Tvergaard–Needleman (GTN) damage model to evaluate the ductile damage behavior of silicon carbide (SiC) reinforced aluminum matrix composites (SiC/Al composites). Uniaxial tensile experiments were conducted at room temperature under controlled strain rates ranging from 0.001 to 0.009 s^−1^. Fracture surface analysis using scanning electron microscopy (SEM) revealed predominantly brittle cleavage features at lower strain rates, with an increasing presence of dimples associated with microvoid coalescence at the highest strain rate, highlighting a strain-rate-dependent fracture mechanism. GTN parameters were determined using finite element simulations combined with response surface methodology (RSM). The results demonstrated that higher strain rates accelerate void nucleation and growth, leading to faster damage evolution. Numerical simulations validated the identified GTN parameters, showing strong agreement with experimental observations. This work provides critical insights into strain-rate effects on damage evolution in SiC/Al composites, supporting their application in high-strain-rate environments.

## 1. Introduction

Silicon carbide-reinforced aluminum matrix composites (SiC/Al composites) are advanced materials that combine the high strength and stiffness of SiC particles with the ductility and low density of aluminum [1,2]. This combination yields superior mechanical performance, including enhanced wear resistance and improved thermal stability, compared to conventional alloys [3,4]. As a result, SiC/Al composites are increasingly used in high-performance automotive and aerospace components. In particular, the automotive industry employs these composites for parts such as brake discs and engine pistons, which must withstand extreme service conditions [5]. These components often experience violent shocks and dynamic loads, under which the material undergoes plastic deformation to absorb impact energy [6].

During such dynamic loading, microvoids nucleate and grow within the ductile aluminum matrix of the composite [7], eventually coalescing and leading to fracture of the material [8]. In essence, the deformation process under impact is a damage degradation process characterized by the continuous nucleation and proliferation of microscopic voids in the matrix [9]. Quantifying the evolution of damage in SiC/Al composites is challenging, however, because this void growth occurs at the microscale and cannot be directly observed in real time. One useful damage metric is the volume fraction of microvoids in the material, which directly reflects the extent of internal damage accumulation [10]. To accurately track this metric during deformation, a reliable damage model that incorporates void evolution is required.

A wide range of damage modeling approaches have been developed, generally classified as uncoupled phenomenological models, coupled phenomenological models, or micromechanical models [11]. Phenomenological models (whether uncoupled or coupled) are often favored in industry for their computational efficiency and simplicity, and they have a higher tolerance for complex engineering simulations. However, the parameters in phenomenological models are usually calibrated to specific experimental conditions, which means these models cannot intrinsically capture the underlying damage mechanisms like microvoid nucleation and growth [12]. In other words, traditional phenomenological models may fit observed behavior under certain conditions but do not fundamentally describe how damage progresses within the material’s microstructure. This limitation makes it difficult for phenomenological models to predict damage evolution outside their calibration range or under varying loading conditions. By contrast, micromechanical damage models explicitly account for the nucleation and growth of voids at the microscale, providing a physics-based representation of material degradation. As a result, micromechanical models address the shortcomings of phenomenological approaches by linking the macroscopic material response to microscopic damage processes [13].

A prominent micromechanical framework for ductile damage is the Gurson–Tvergaard–Needleman (GTN) model, which incorporates the effect of the void volume fraction on the material’s yield behavior. The GTN model was originally proposed by Gurson and later refined by Tvergaard and Needleman to better predict void growth and coalescence in a deforming metal [14]. In this model, the presence and evolution of microscopic voids are directly coupled with plastic flow: the growth of microvoids reduces the load-bearing cross-section of the material, which in turn alters the yield surface, and conversely, continued plastic deformation accelerates void growth. Modern finite element implementations of the GTN model can thus track the volume fraction of microvoids in real time during a simulated deformation process, making it an attractive tool for evaluating damage accumulation. Over the past decades, the GTN model has been widely applied to simulate ductile fracture in metals and alloys. It is now recognized as an effective approach for describing damage evolution in a variety of materials, including high-strength steels, aluminum alloys, shape-memory alloys, and titanium alloys [15]. For example, Semiatin et al. [16] and Dong et al. [17] employed a GTN-based analysis to investigate void growth (cavitation) in Ti–6Al–4V titanium alloys during high-temperature tensile deformation, successfully capturing the damage evolution under different temperatures. However, despite its broad success in metals, the application of the GTN model to particle-reinforced metal matrix composites like SiC/Al remains underexplored, particularly for varying strain rates and complex stress states. It is worth noting that in SiC/Al composites, ductile failure is predominantly governed by void nucleation and growth in the aluminum matrix, while the SiC particles may fracture or debond in a brittle manner. Therefore, a void-based damage model like GTN is expected to be applicable for capturing the matrix-dominated failure process, provided that its parameters are properly calibrated for the composite’s heterogeneous microstructure.

Only a few recent studies have begun to apply GTN-type models to SiC/Al composites. For instance, Ma et al. [5] used a GTN damage framework to simulate the tensile fracture behavior of SiC_p/Al composites containing 10–14% SiC particles and reported good agreement between the model predictions and experimental stress–strain responses. Another study developed a modified GTN-based microscale model (incorporating particle fracture and interface debonding) for SiC_p/Al composites and demonstrated that the reinforcement particle size and morphology have a significant effect on the composite’s failure behavior [18]. These efforts underscore the potential of micromechanical damage modeling for SiC/Al composites. Nevertheless, a systematic calibration and validation of GTN model parameters for SiC/Al composites under different loading rates is still lacking in the literature. In particular, the influence of strain rate on void nucleation and growth in these composites has not been thoroughly investigated using the GTN approach, leaving a critical gap in our ability to predict rate-dependent failure in SiC/Al components.

In this study, we address the above gap by calibrating and validating a GTN damage model for SiC/Al composites through a combined experimental–numerical approach. Quasi-static uniaxial tensile tests were performed on SiC/Al composite specimens at strain rates ranging from 0.001 s^−1^ to 0.009 s^−1^ in order to generate stress–strain data and observe damage progression for model calibration. The key GTN model parameters (related to void nucleation, growth, and coalescence) were then optimized using a response surface methodology (RSM), with the goal of reproducing the experimentally measured mechanical behavior. With the calibrated parameters, finite element simulations were conducted to verify the GTN model’s predictive capability by comparing the simulated results against experimental observations. Through this hybrid calibration and validation process, the evolution of microvoid volume fraction—as an indicator of damage degradation—can be dynamically evaluated for the SiC/Al composite under different loading conditions. The outcomes of this study provide a deeper insight into the damage mechanisms of SiC/Al composites and establish a reliable modeling framework for predicting their failure behavior across various strain rates.

## 2. Model and Parameter Calibration

### 2.1. GTN Framework

**Figure 1:**
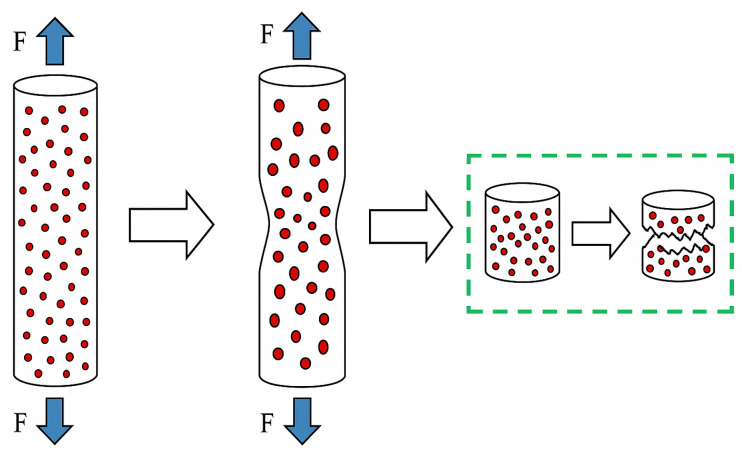
Schematic illustration of ductile damage process of alloys.

The GTN model is a micromechanical approach that accounts for the influence of microstructural ductile damage on material-yielding behavior [5]. Unlike the classical plasticity criterion, the yield surface in the GTN model incorporates hydrostatic stress dependence, reflecting strain softening or hardening caused by the presence of voids in the material. The evolution of ductile damage in alloys during deformation can be broadly divided into four stages: nucleation of voids near inclusions or defects, growth of voids driven by subsequent plastic deformation, coalescence of voids leading to micro-crack formation and, eventually, the development of macro-cracks through the merging of micro-cracks. This process is illustrated schematically in Figure 1.

**Figure 2:**
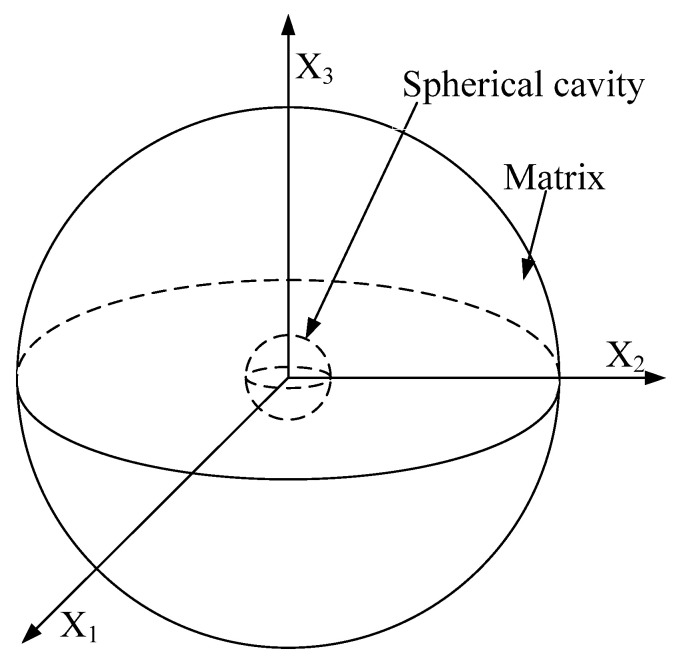
Model of the hollow sphere.

The influence of microvoid evolution mechanisms on yielding behavior is included by modifying the isotropic Von Mises yield criterion. Figure 2 shows the yield potential for a spherical body made of a ductile matrix material with a spherical void. The yield function for the damaged material, which incorporates the effects of microvoid evolution, is expressed as follows [19]:

(1) $$\Phi ={\left(\frac{\bar{\sigma }}{\sigma_{y}}\right)}^{2}+f^{*}\left(2Q_{1}cosh\left(-\frac{3}{2}Q_{2}\frac{\sigma_{m}}{\sigma_{y}}\right)-qQ_{3}\right)-1=0$$

where *Q*1, *Q*2, and *Q*3 represent the modified material parameters used to describe the influence of void interaction and stress field inhomogeneity around microvoids. In this study, *Q*1 = 1.5, *Q*2 = 1.0, and *Q*3 = 2.25 are adopted following the classical Tvergaard–Needleman recommendation. These parameters were originally calibrated through detailed unit cell analyses and have been widely applied across a broad range of ductile metallic materials. Although reference involves a different alloy system, these q-parameters are generally regarded as weakly material-dependent correction factors associated with void interaction effects rather than specific alloy chemistry. Therefore, their use in the present SiC/Al composite is considered reasonable and consistent with common GTN modeling practice. $\sigma_{m}$ represents the hydrostatic stress; $\sigma_{y}$ represents the yield stress of matrix; $\bar{\sigma }$ represents the Von Mises equivalent stress; and the symbol $f^{*}$ represents the loss of stress because of void coalescence, as calibrated in Equation (2) [19]:

(2) $$f^{*}=\left\{\begin{array}{cc} f & \mathrm{if}\;f\leq f_{c}\\ f_{c}+\frac{f_{u}^{*}-f_{c}}{f_{f}-f_{c}}(f-f_{c}) & \mathrm{if}\;f>f_{c} \end{array}\right.$$

where f is the volume fraction of void; $f_{u}^{*}$ represents the disappearance of the stress carrying capacity, which can be calculated by the formula: $f_{u}^{*}=\frac{1}{q_{1}}$; $f_{f}$ is the final void volume fraction under the condition of complete stress loss; $f_{c}$ is the critical volume fraction at the onset of microvoid nucleation and coalescence.

The increase of the microvoid volume fraction originated from the contributions of new nucleation of voids ${\dot{f}}_{nucl.}$ and the growth of previous voids ${\dot{f}}_{grow}$; the function can be expressed as follows [19]:

(3) $$\left\{\begin{array}{l} \dot{f}={\dot{f}}_{nucl.}+{\dot{f}}_{grow}\\ {\dot{f}}_{nucl.}=A{\bar{\varepsilon }}^{p}\\ {\dot{f}}_{grow}=(1-f)\varepsilon_{kk}^{p} \end{array}\right.$$

where $\varepsilon_{kk}^{p}$ represents the plastic hydrostatic strain, ${\bar{\varepsilon }}_{p}$ represents the equivalent plastic strain, and the coefficient is A. The following formula is given for A [20]:

(4) $$A=\frac{f_{N}}{S_{N}\cdot \sqrt{2\pi }}\cdot \exp\left[-\frac{1}{2}\cdot (\frac{{\bar{\varepsilon }}_{p}-\varepsilon_{N}}{S_{N}}{)}^{2}\right]$$

where $f_{N}$ represents the volume fraction of microvoid nucleation, $S_{N}$ represents the standard deviation, and $\varepsilon_{N}$ represents the mean effective strain of microvoid nucleation. According to the reference, the values of $\varepsilon_{N}$ and $S_{N}$ can be taken as 0.3 and 0.1, respectively.

The GTN model can effectively characterize the damage evolution of SiC-reinforced Al matrix composites. However, determining the parameters of the GTN model remains a challenging task. Accurate identification of the GTN parameters—such as the initial void volume fraction (*f*_0_), critical void volume fraction (*f_c_*), nucleation void volume fraction (*f_n_*), and final void volume fraction (*f_f_*)—is essential for adequately capturing the ductile damage process. Various approaches have been employed to solve for these parameters. Additionally, unit cell model simulations have been used in several studies to obtain these parameters. Furthermore, inverse methods, which rely on numerical simulations, have been employed to determine the GTN model parameters in some works [21]. These methods generally involve comparing numerical results with experimental data and using nonlinear least squares fitting to choose the most suitable parameters. Consequently, a well-structured experimental design is crucial for accurately identifying the GTN model parameters. Response Surface Methodology (RSM) is particularly effective in this regard, as it can account for multiple factors at various levels and establish a functional relationship between the factors and response variables [22].

### 2.2. RSM-Based Parameter Identification

The Response Surface Method (RSM) is a statistical approach to optimizing stochastic processes [23]. It aims to establish quantitative relationships between the test indicators and influencing factors through nonlinear regression fitting. This method closely approximates the complex multi-dimensional space to real-world conditions while requiring fewer test groups. Experimental data from varying factors can be used to derive the response surface values. The functional relationship between response indicators (*Y*) and variables (*X_i_*) can be expressed by the following formula [24]:

*Y = f* (*x*_1_, *x*_2_, ⋯, *x_n_*) (5)

where *X* represents the values of the variables, *Y* denotes the values of the indicators, and *n* refers to the number of variables.

Typically, the functional relationships between the indicators and variables are either unknown or complex. Therefore, selecting an appropriate function is essential for obtaining an accurate regression equation. To balance the time required with the simplicity of the fitting equations, linear or quadratic polynomial functions are commonly used to derive the regression equation.

## 3. Experimental Procedures

### 3.1. Material and Tensile Testing

The SiC/Al composite, consisting of 8.75 wt.% SiC with the balance consisting of Al (Table 1) was machined into tensile specimens according to the ASTM E8 standard [19] for tension testing of metallic materials (Figure 1). Tensile tests were performed using an ETM105 servo-hydraulic testing machine (Wance Testing Machine Co., Ltd., Shenzhen, China) at strain rates ranging from 0.001 to 0.009 s^−1^. Load-displacement data were recorded until fracture.

Load-displacement data must be acquired first to solve the GTN parameters using the inverse method. Therefore, a series of uniaxial tensile tests, conducted under different strain rates at room temperature, must be performed on Silicon Carbide-reinforced Aluminum Matrix Composites (SiC-AMCs). The as-received material consists of a block of as-cast SiC-AMCs, with the chemical compositions determined using an optical emission spectrometer (OES) or energy-dispersive X-ray spectroscopy (EDS), as outlined in Table 1.

**Figure 3:**
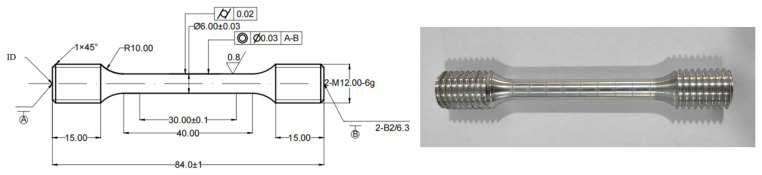
The shape and size of the tensile samples.

The uniaxial tensile experiments were designed in accordance with the ASTM E8/E8M-24 standard for tension testing of metallic materials [25]. The geometry and dimensions of the tensile specimens are shown in Figure 3 (dimensions in millimeters). Four specimens were extracted from the as-cast block using wire electrical discharge machining (EDM). Prior to testing, all specimen surfaces were ground to remove surface defects. The tests were conducted using a computer-controlled, servo-hydraulic tensile testing machine (ETM105 SYS-145), with the specimens clamped by threaded connections. The tensile speed was set via the control system, initiating the test process.

Tensile speeds of 2.4, 7.2, 12, 16.8, and 21.6 mm/min corresponded to accurate strain rates of 0.001, 0.003, 0.005, 0.007, and 0.009 s^−1^, respectively. These low strain rates were chosen to represent quasi-static loading and to avoid significant inertial effects, ensuring the accurate characterization of fracture behavior [26]. Each tensile test was repeated four times at room temperature under the specified strain rates. All specimens were pulled to complete failure. Throughout the tests, a real-time data acquisition system continuously recorded load and displacement.

### 3.2. Fractography and Stress–Strain Analysis

**Figure 4:**
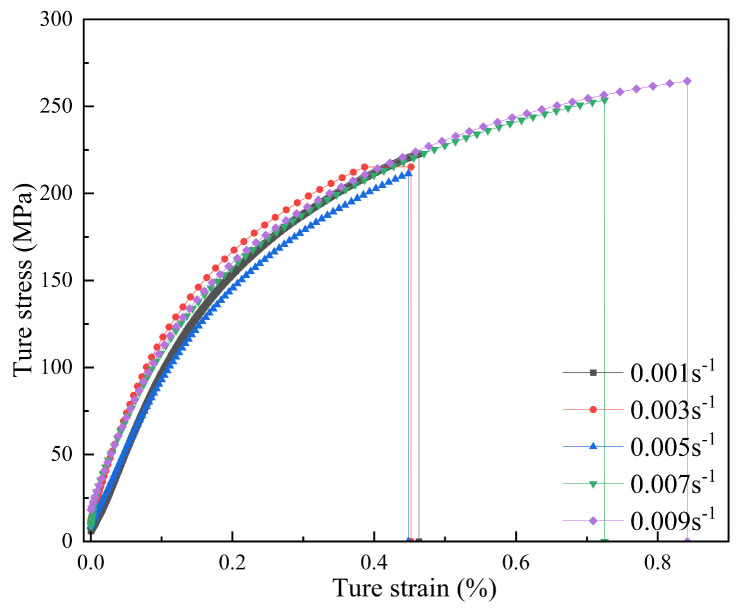
True stress–strain curves of SiC/Al composites obtained by tensile tests at room temperature with different strain rates.

According to the load–distance data, the nominal stress–strain can be obtained by $\sigma_{N}=F/S$, $\varepsilon_{N}={\;d/D}_{0}$ where $\sigma_{N}$ represents the nominal strain; F is the load; S is the cross sectional area of samples; $\varepsilon_{N}$ is the nominal strain; *d* is the recorded distance; *D*_0_ is the initial distance. Further, the nominal stress–strain data were converted into true stress–strain data based on the formula $\sigma_{T}=\sigma_{N}\left(1+\varepsilon_{N}\right)$, $\varepsilon_{T}=\;\ln\left(1+\varepsilon_{N}\right)$, where $\sigma_{T}$ is the true stress and $\varepsilon_{T}$ is the true strain [27,28]. Figure 4 presents the actual stress–accurate strain curves obtained from tensile tests conducted at different strain rates, ranging from 0.001 s^−1^ to 0.009 s^−1^. The results demonstrate that the strain rate significantly affects the material’s mechanical response. The material exhibits lower flow stress at lower strain rates (0.001 s^−1^ to 0.005 s^−1^), whereas at higher strain rates (0.007 s^−1^ and 0.009 s^−1^), the flow stress increases, enhancing the material’s strength. Furthermore, the fracture strain shows a non-monotonic trend. At lower strain rates, the material undergoes more significant plastic deformation before failure, while at 0.007 s^−1^ and 0.009 s^−1^, the fracture strain increases significantly. The findings suggest that the material retains substantial ductility at high strain rates, even as its strength increases. This observed trend can be attributed to the interplay between strain hardening and the inhibition of dislocation movement under high strain rate conditions. Overall, the results indicate that the material’s mechanical behavior depends on the strain rate, with both strength and ductility significantly influenced by the loading conditions.

**Figure 5:**
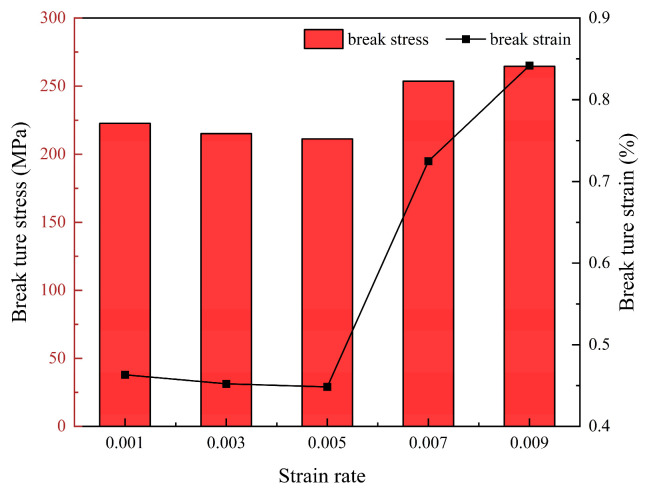
The statistics of the break stress and break strain were obtained from the tensile experiments at room temperature with different strain rates.

The flow stress of the SiC/Al composite exhibits high sensitivity to strain rate. Key data points such as fracture stress and strain were extracted from the flow curves, as shown in Figure 5, to illustrate the relationship between flow stress and strain rate. The results indicate that strain rate significantly affects both fracture stress and fracture strain. Specifically, as the strain rate increases from 0.001 s^−1^ to 0.009 s^−1^, the fracture stress generally increases, with a notable rise at 0.007 s^−1^ and above, suggesting an enhancement in the material’s load-bearing capacity at higher strain rates. This behavior may be attributed to the suppression of dislocation motion, leading to a strengthening effect.

In contrast, fracture strain remains relatively low at 0.001–0.005 s^−1^ but increases sharply at 0.007 s^−1^ and 0.009 s^−1^, indicating more excellent deformability at higher strain rates. This phenomenon can be explained by the increased local stress concentration and changes in plastic deformation mechanisms under high-strain-rate conditions. Additionally, voids have insufficient time to coalesce at higher strain rates, reducing the fracture strain. Overall, the fracture behavior of the SiC/Al composite is strongly influenced by strain rate. At higher strain rates, both the material’s load-bearing capacity and plasticity are enhanced. It should be clarified that the increase in fracture strain at higher strain rates observed in Figure 5 indicates enhanced macroscopic deformation capacity rather than a fully ductile fracture mechanism. For particle-reinforced SiC/Al composites, final failure is still primarily governed by brittle mechanisms such as SiC particle fracture and particle–matrix interface debonding. Therefore, a higher global fracture strain does not necessarily imply a transition to classic ductile dimple fracture at the microscale.

### 3.3. Fracture Morphology Observation

Fracture surface morphologies were examined using a high-resolution scanning electron microscope (JEOL SEM) operated at an accelerating voltage of 20.0 kV with a working distance of approximately 20.0 mm. To reduce surface charging and enhance image clarity, all specimens were sputter-coated with a thin conductive gold layer prior to imaging. The secondary electron (SE) mode was employed to resolve the topographical features of the fracture surfaces. Specimens were sectioned from approximately 2 mm below the tensile fracture surface to ensure representative microstructural analysis. Observations were conducted at a magnification of ×1000 to assess fracture characteristics, including cleavage steps, crack propagation behavior, and evidence of localized plastic deformation.

**Figure 6:**
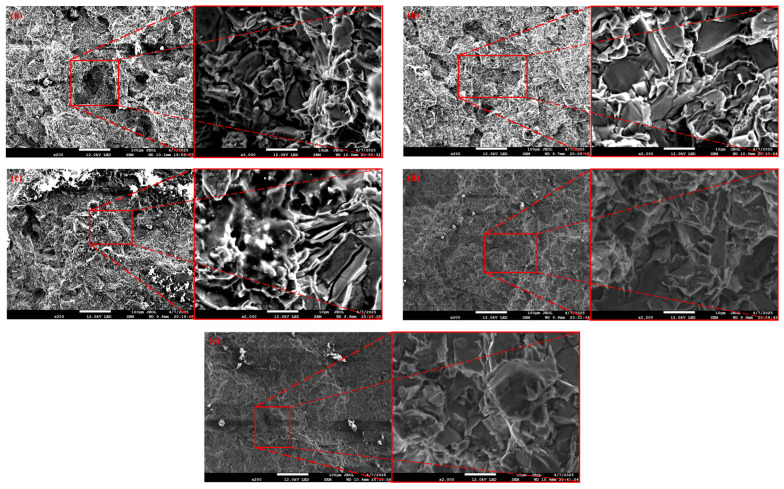
SEM images of the fracture surfaces of SiC/Al composites at different strain rates. (**a**) 0.001 s^−1^, (**b**) 0.003 s^−1^, (**c**) 0.005 s^−1^, (**d**) 0.007 s^−1^, and (**e**) 0.009 s^−1^.

As shown in Figure 6a–e, the tensile fracture surfaces of the material exhibit morphological variations as a function of applied strain rate, ranging from 0.001 s^−1^ to 0.009 s^−1^ in five equal increments. All specimens predominantly display features characteristic of brittle fracture, including flat cleavage planes and sharp intergranular steps, which are particularly evident in Figure 6a (0.001 s^−1^) and Figure 6d (0.007 s^−1^). This observation does not contradict the higher fracture strain measured at elevated strain rates. In SiC/Al composites, fracture mode is controlled by the brittle ceramic reinforcement and interface strength, while the aluminum matrix mainly contributes to plastic accommodation before final failure. As a result, the composite may exhibit increased overall elongation under higher strain rate loading yet still present predominantly cleavage- and particle-controlled brittle features on the fracture surface. This macro–micro response decoupling is typical for particulate metal matrix composites. These features indicate transgranular fracture along crystallographic cleavage paths, typical of high-strength, low-toughness materials.

However, as the strain rate increases, subtle changes in the fracture topography emerge. At intermediate rates (e.g., 0.003 s^−1^ in Figure 6b and 0.005 s^−1^ in Figure 6e), the surfaces exhibit more rugged morphologies with irregular tearing ridges and isolated shallow dimples, suggesting the activation of limited plastic deformation mechanisms in localized zones. Most notably, at the highest strain rate (0.009 s^−1^, Figure 6c), the fracture surface becomes significantly rougher, displaying microvoid coalescence and ductile tearing features embedded within the predominantly brittle matrix.

These results indicate that while the composite’s fracture mode is intrinsically brittle, a non-negligible degree of ductile behavior is activated at higher strain rates due to localized stress intensification and energy dissipation in the matrix. The presence of microvoids and tearing ridges at elevated rates implies that the aluminum matrix undergoes more plastic deformation before fracture when the loading is faster. This strain-rate-sensitive mix of brittle and ductile features likely arises from the composite microstructure: the rigid SiC particles promote cleavage and cracking at low rates, but at higher rates the ductile aluminum matrix can accommodate more deformation (via void nucleation and slip), partially blunting cracks. In particular, the pronounced microvoid coalescence observed at 0.009 s^−1^ corresponds to the increased fracture strain measured at this highest rate, suggesting that the matrix underwent additional void growth and plastic deformation before final failure. At lower strain rates (e.g., 0.001 s^−1^), by contrast, fracture occurs with minimal void growth (cleavage-dominated), which is consistent with the much lower fracture strain. Overall, these fractographic observations suggest that fracture in the SiC/Al composite initiates and progresses primarily through void formation in the ductile aluminum matrix (rather than catastrophic particle breakage), which supports the use of the GTN model focusing on ductile matrix damage.

## 4. Results and Discussion

### 4.1. Numerical Modeling of the Uniaxial Tensile Test

**Figure 7:**
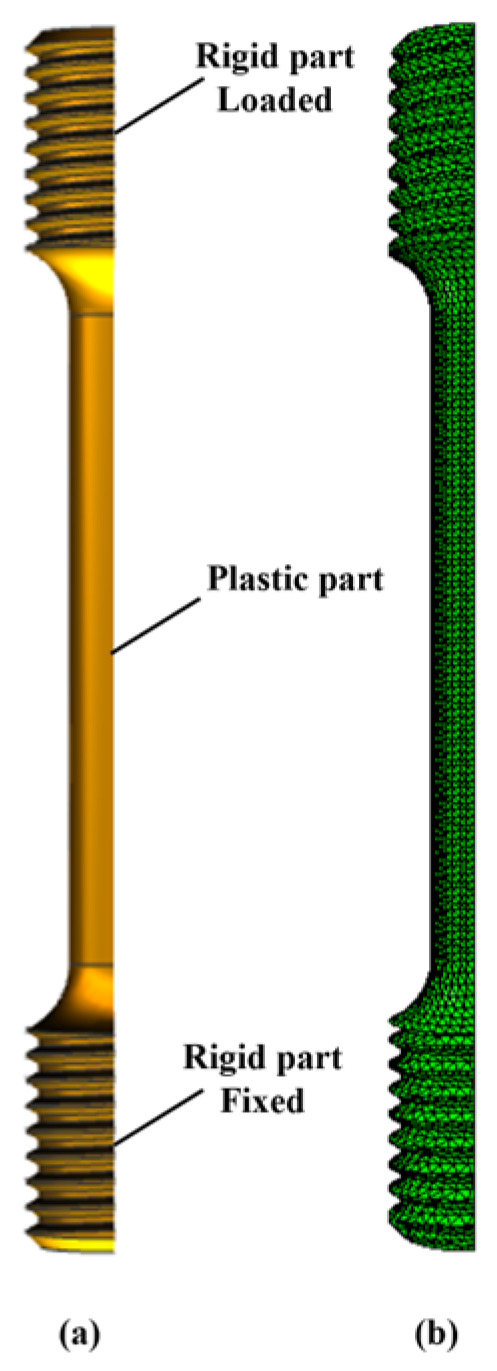
(**a**) Axisymmetric model of the uniaxial tensile test; (**b**) finite element model with mesh.

**Figure 8:**
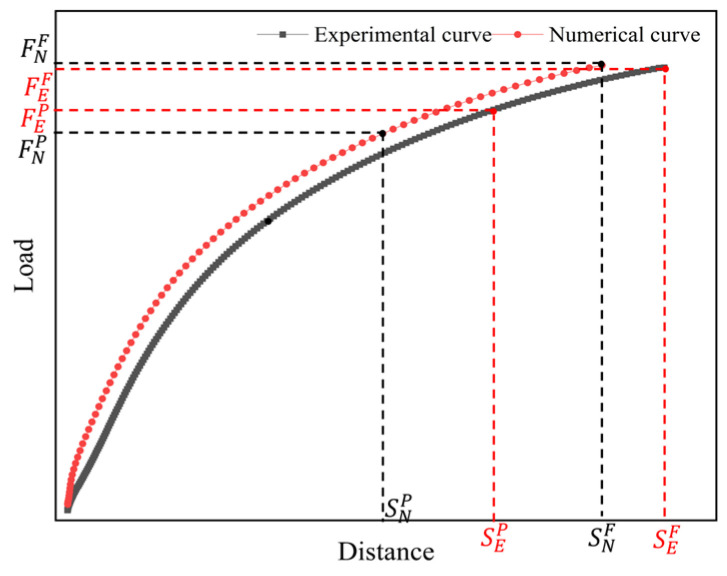
Representation of four reference points to obtain responses.

The finite element simulation of uniaxial tensile tests aims to generate load–distance curves, which are then compared with experimental data to determine the most suitable GTN parameters. To reduce simulation time, the tensile test model was simplified to an axisymmetric configuration, as shown in Figure 7. The sample was discretized using four-node bilinear axisymmetric quadrilateral elements with reduced integration and hourglass control (CAX4R). Both ends of the sample were constrained as rigid parts and connected to corresponding reference points to simulate the tensile process and improve convergence rates. The load was applied to the reference points at a consistent tensile speed. Explicit dynamic analysis was employed for the simulations; the required material parameters are provided in Table 2. To ensure that using an explicit solver did not introduce inertial effects in this quasi-static simulation, the loading rate was kept very low and the model’s kinetic energy was monitored to remain below 5% of the internal energy. This verified that the simulation conditions were quasi-static, thus validating the use of the explicit dynamic approach. To improve the transparency of the numerical model, an enlarged mesh view was added in the revised Figure 8 to explicitly show element shape, size distribution, and local mesh density. The model employed structured axisymmetric quadrilateral elements, with local refinement in the gauge and necking regions to ensure damage evolution accuracy. Mesh sensitivity checks confirmed that further refinement produced negligible change in the load–displacement response.

### 4.2. Experimental Design for RSM

The GTN parameters to be identified include the initial void volume fraction (*f*_0_), critical void volume fraction (*f_c_*), nucleation void volume fraction (*f_n_*), and final void volume fraction (*f_f_*). It should be noted that the void volume fraction parameters reported in Refs. [29,30] were originally calibrated for Ti–6Al–4V alloy. In the absence of directly measured GTN parameters for SiC/Al composites, these values were used as physically reasonable initial estimates because they fall within the same order of magnitude as the reported GTN parameters for aluminum alloys. More importantly, in the present work, these parameters were not directly adopted but were further optimized through response surface methodology (RSM) inverse identification based on experimental tensile curves. Therefore, the final parameter set reflects experimental calibration for the current composite rather than direct transfer from titanium alloy data [24]. A central composite design (CCD) was employed to plan the simulations, owing to its proven effectiveness in exploring a multi-factor parameter space with a limited number of runs. Twenty-five uniaxial tensile simulations were conducted at each strain rate, as outlined in Table 3. As illustrated in Figure 8, four reference points on the load–distance curves were chosen to determine the response values and assess the accuracy of the simulated results against the experimental data. The response values, denoted as *R*, are the differences between the corresponding reference points. The specific formulation of these response values is provided below.

(6) $$\left\{\begin{array}{c} R_{1}=S_{E}^{P}-S_{N}^{P}\\ R_{2}=F_{E}^{P}-F_{N}^{P}\\ R_{3}=S_{E}^{F}-S_{N}^{F}\\ R_{4}=F_{E}^{F}-F_{N}^{F} \end{array}\right.$$

where $S_{E}^{P}$ and $S_{N}^{P}$ represent the distances at maximum load on the experimental curves and numerical curves, respectively; $F_{E}^{P}$ and $F_{N}^{P}$ represent the maximum load of the experimental curves and numerical curves, respectively; $S_{E}^{F}$ and $S_{N}^{F}$ represent the distance at the breakpoints of experimental curves and numerical curves, respectively, $F_{E}^{F}$ and $F_{N}^{F}$ represent the load at the breakpoints of the experimental curve and numerical curve, respectively. In order to promote the accuracy of the identified parameters, the corresponding relationships between factors and response values were fitted by the quadratic polynomial function, and the expression is shown as follows [31,32]:

(7) $$R=b_{0}+\sum_{i=1}^{4}b_{i}X_{i}+\sum_{i=1}^{4}b_{ii}X_{i}^{2}+\sum_{i=1}^{3}\sum_{j<i}^{4}b_{ij}X_{i}X_{j}$$

where R represents the response values, including $R_{1}$, $R_{2}$, $R_{3}$; $R_{4}$, $b_{0}$, $b_{i}$, $b_{ii}$ and $b_{ij}$ are the coefficients of the constant, linear, interaction and quadratic terms, respectively; $X_{i}$ and $X_{j}$ represent the four factors, i.e., $f_{0}$, $f_{N}$, $f_{c}$ and $f_{f}$. Subsequently, the desired values for the four factors can be obtained using the least squares method in MATLAB R2022b (MathWorks Inc., Natick, MA, USA). In this way, the optimal set of GTN model parameters for each strain rate was obtained through the RSM optimization procedure.

### 4.3. GTN Parameters of SiC/Al Composites at Different Strain Rates

The response values for various factor levels were calculated based on the procedure outlined earlier, and the results are provided in Table 3. The final response functions for a strain rate of 0.001 s^−1^ are presented below:

$$\begin{array}{l} R_{1}=6.04-84.54352\times f_{0}-108.93191\times f_{n}-7.42985\times f_{c}+9.84\times f_{f}-209.54375\times f_{0}\times f_{n}\\ -655.75\times f_{0}\times f_{c}+42.64\times f_{0}\times f_{f}-19.86\times f_{n}\times f_{c}-26.03\times f_{n}\times f_{f}+54.078\times f_{c}\times f_{f}\\ +8708.40636\times {f_{0}}^{2}+990.56656\times {f_{n}}^{2}+12.53972\times {f_{c}}^{2}-32.77734\times {f_{f}}^{2}\\ R_{2}=775.71081+22835.47552\times f_{0}+11.88677\times f_{n}+1.9\times f_{c}+0.32\times f_{f}-1875\times f_{0}\times f_{n}\\ -1977.40113\times {f_{0}}^{2}-19.77401\times {f_{n}}^{2}-12.65537\times {f_{c}}^{2}-0.79096\times {f_{f}}^{2}\\ R_{3}=3.45709+427.42723\times f_{0}+58.95888\times f_{n}+18.35\times f_{c}-18.72912\times f_{f}-329.5\times f_{0}\times f_{n}\\ -1124.6\times f_{0}\times f_{c}-440.85\times f_{0}\times f_{f}-85.455\times f_{n}\times f_{c}+4.44\times f_{n}\times f_{f}+66.121\times f_{c}\times f_{f}\\ -38822.31638\times {f_{0}}^{2}-267.24816\times {f_{n}}^{2}-199.46282\times {f_{c}}^{2}+27.88357\times {f_{f}}^{2}\\ R_{4}=3499.33761-4.64614E6\times f_{0}+6.09871E5\times f_{n}-7.30245E5\times f_{c}+1.57867E5\times f_{f}\\ +1.1E7\times f_{0}\times f_{n}+6.3E6\times f_{0}\times f_{c}+4.1E6\times f_{0}\times f_{f}+1.3E6\times f_{n}\times f_{c}+4E5\times f_{n}\times f_{f}\\ -4.5E5\times f_{c}\times f_{f}+4.94E8\times {f_{0}}^{2}-6.67E6\times {f_{n}}^{2}+4.67E6\times {f_{c}}^{2}-3.43E5\times {f_{f}}^{2} \end{array}$$

Subsequently, the response functions were solved using Matlab software to determine the optimal values of the independent variables, i.e., the most suitable GTN parameters: *f*_0_ = 0.005, *f_n_* = 0.0412, *f_c_* = 0.0686 and *f_f_* = 0.3016 [27,28]. Using the RSM optimization approach, we determined the GTN damage model parameters that best matched the experimental tensile curves at each strain rate. The optimized parameter values are listed in Table 4. It should be emphasized that although the initial parameter ranges were informed by the literature values from other alloys, the final GTN parameters reported in Table 4 were obtained through inverse calibration against the present experimental tensile data using RSM optimization. Therefore, the reported parameters are experiment-consistent values for the SiC/Al composite studied here rather than direct literature transplants.

The strain rate significantly affects the GTN parameters, especially the void volume fractions *f_c_* and *f_f_*. As the strain rate increases from 0.001 to 0.009 s^−1^, the final void volume fraction (*f_f_*) decreases markedly, while the void nucleation fraction (*f_N_*) and critical void volume fraction (*f_c_*) both increase. The initial void volume fraction (*f*_0_) remains essentially unchanged, as expected for specimens taken from the same billet (indicating similar initial porosity). Physically, these trends can be understood as follows: at higher strain rates, void nucleation is more pronounced (higher *f_N_*) due to greater stress concentrations at particle–matrix interfaces under rapid loading. The critical void fraction *f_c_* is also higher at high rates, suggesting the material can tolerate a larger volume fraction of damage before triggering rapid coalescence—possibly because multiple voids form and grow concurrently, delaying the onset of coalescence. However, the final void fraction *f_f_* is much lower at high rates, meaning the composite fractures at a lower overall void content; this occurs because there is insufficient time for voids to grow and merge into larger cavities before failure. These parameter variations are consistent with our fractography observations: for example, at 0.009 s^−1^ the fracture surface dimples are small and shallow (voids did not have time to grow large before fracture), whereas at 0.001 s^−1^ the dimples are larger and deeper, reflecting more extensive void growth. In summary, the identified GTN parameters capture the strain-rate-dependent void nucleation and growth behavior in the composite’s microstructure.

### 4.4. Evaluation of the Damage Degradation Process for SiC/Al Composites

**Figure 9:**
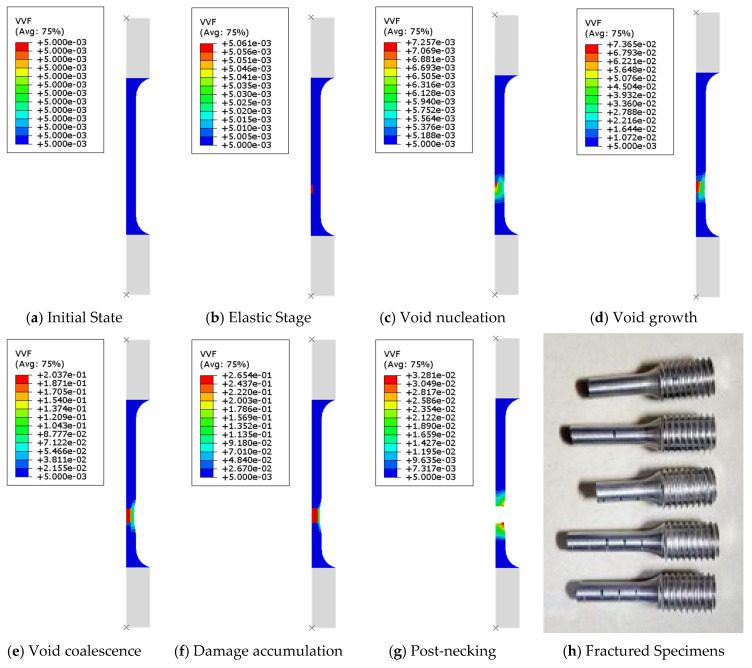
Distribution and evolution of void volume fraction in the specimen during tensile deformation: (**a**) initial state, (**b**) elastic stage, (**c**) void nucleation, (**d**) void growth, (**e**) void coalescence, (**f**) damage accumulation, (**g**) post-necking (just before fracture), and (**h**) photograph of the fractured specimen.

To investigate the damage evolution behavior of silicon carbide-reinforced aluminum matrix composites under tensile loading, a finite element simulation was performed using the calibrated GTN model parameters. The simulation was conducted at a quasi-static strain rate of 0.001 s^−1^, and the results are illustrated in Figure 9, which presents the distribution and progressive evolution of void volume fraction (VVF) throughout the tensile process [28].

As shown in Figure 9a–g, the VVF remains negligible during the initial elastic stage (Figure 9a,b). As plastic deformation proceeds, microvoids begin to nucleate in localized stress concentration regions (Figure 9c), followed by growth in size and number (Figure 9d). These voids gradually coalesce (Figure 9e,f), leading to pronounced damage accumulation in the necking region (Figure 9g). The final VVF exceeds 0.3 and is predominantly concentrated around the predicted fracture zone, indicating severe material degradation. Figure 9h shows the actual fractured specimens, and the experimentally observed fracture location closely corresponds with the simulated damage localization. This strong consistency validates the reliability of the GTN model in describing the damage evolution of SiC/Al composites under low-strain-rate tensile loading.

**Figure 10:**
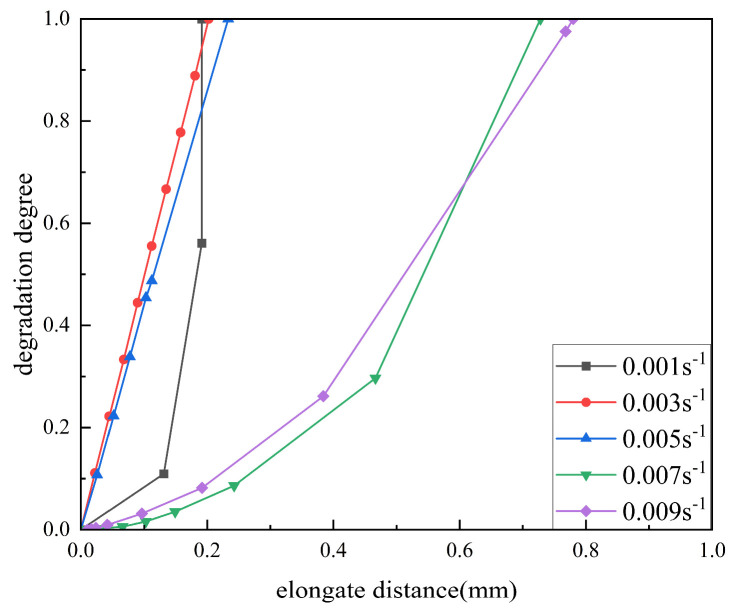
Damage degradation (void accumulation) as a function of strain for various strain rates.

By tracking the evolution of the microvoid volume fraction during deformation, we can quantify a “damage degradation degree”—defined here as the ratio of the void volume at a given strain to the void volume at final fracture. In our case, the maximum degradation degree (at fracture) corresponds to a void volume fraction of ~0.85 (or 85% of the cross-section voided). Using this metric, damage–strain curves were constructed for each strain rate, as presented in Figure 10. These curves illustrate how damage accumulates (in terms of increasing void fraction) as a function of tensile strain for the different strain rates.

Figure 10 reveals that strain rate has a profound effect on the damage accumulation behavior. At low strain rates (0.001–0.003 s^−1^), the material is highly sensitive to damage: once the void volume fraction reaches around 0.4 (40% damage), the remaining elongation drops sharply (from ~0.8 mm at near-zero damage to ~0.2 mm at 40% damage). This indicates that under slow loading, even a moderate amount of microvoid growth leads to a large reduction in ductility, since voids have ample time to grow and coalesce, rapidly compromising the load-bearing area. At intermediate strain rates (0.005–0.007 s^−1^), the influence of damage on mechanical response becomes more gradual, until a critical threshold is reached. In this range, there appears to be a brittle-to-ductile transition: below a certain damage level (~20% void fraction), the composite retains relatively high ductility; however, once damage exceeds that ~0.2 threshold, the elongation to failure drops abruptly (by roughly 40%). This threshold behavior suggests that at moderate rates the matrix can accommodate void growth up to a point, after which crack coalescence accelerates and causes sudden failure. At the highest strain rate (0.009 s^−1^), an anomalous behavior is observed: despite significant damage accumulation, the composite still achieves an elongation of about 0.8 mm, similar to that at the lowest rate. This unexpectedly high ductility under fast loading may be due to dynamic effects such as localized thermal softening or delayed crack propagation, which allow additional plastic deformation before fracture. In essence, the rapid loading might activate an energy dissipation mechanism that temporarily enhances the material’s ductility, counteracting the tendency toward brittleness.

These experimental findings also shed light on the GTN model’s applicability and limitations. The existence of a strain-rate-dependent brittle-to-ductile transition and the role of stress concentration effects imply that GTN model parameters should be considered as functions of strain rate for best accuracy. The enhanced ductility observed at 0.009 s^−1^ suggests that additional mechanisms (e.g., adiabatic heating or rate-dependent toughening in the matrix) may come into play at high rates—mechanisms that are not explicitly captured by the standard GTN model. While our calibrated GTN model successfully describes the void-driven damage process in the quasi-static regime, it does not account for possible SiC particle fracture or interface debonding, nor for inertial/thermal effects at higher rates. Recognizing these factors provides a theoretical basis for future improvements: the damage evolution parameters of the GTN model could be optimized with input from these strain-rate-sensitive phenomena, and the model could be extended to more accurately describe deformation and failure under dynamic loading conditions.

## 5. Conclusions

A series of uniaxial tensile tests were conducted at strain rates of 0.001 s^−1^ and 0.009 s^−1^ under controlled room temperature conditions to investigate the strain-rate-dependent damage evolution in silicon carbide-reinforced aluminum matrix composites. Based on the combined experimental and numerical results, the following conclusions can be drawn:

(1) A response surface methodology (RSM) framework was established to quantitatively identify the GTN damage parameters of SiC/Al composites under various strain-rate conditions. This approach systematically revealed the strain-rate-dependent variations in GTN parameters, notably the significant increases in critical void volume fraction and void nucleation rate at higher strain rates. The identified parameter set offers a robust basis for constructing rate-sensitive constitutive models (see Table 4).

(2) The GTN parameters exhibited marked sensitivity to strain rate. With increasing strain rate, both the critical void volume fraction and the void nucleation rate rose, indicating earlier and more rapid initiation of damage. Conversely, the final void volume fraction decreased, suggesting that under high loading rates, microvoids lacked sufficient time to evolve into macroscopic damage prior to fracture.

(3) Analysis of damage evolution revealed that elevated strain rates accelerate microvoid nucleation and growth, resulting in premature failure accompanied by limited global plastic deformation. This behavior is consistent with the observed strain-rate-induced changes in GTN parameters and is reflected in the fracture surface morphology (more brittle features at lower rates and more micro-dimples at higher rates). These findings underscore the necessity of incorporating rate-dependent damage models to accurately characterize the mechanical response of such materials.

(4) The GTN model developed in this work effectively captures matrix-void-controlled damage evolution under quasi-static tensile loading. However, the model does not explicitly include SiC particle fracture or particle–matrix interface decohesion mechanisms. These brittle micro-failure modes may also contribute to final fracture in SiC/Al composites. Therefore, the present model should be regarded as a matrix-damage-dominated approximation, and future work should incorporate particle-level failure mechanisms for improved predictive capability.

## Figures and Tables

**Table 1:** Chemical composition of the SiC/Al composite (wt.%).

Element	Si	Fe	Cu	Mg	Ti	All Other Elements	Al
Content (wt.%)	8.75	0.18	0.15	0.55	0.18	0.03 max 0.10 total	Bal.

**Table 2:** The required simulation parameter for SiC/Al composites.

Mass Density Kg/mm^3^	Young’s Modulus Gpa	Poisson’s Ratio	Yield Strength Mpa	Breaking Strength Mpa
2700	75	0.33	225	330

Note: The Young’s modulus (~75 Gpa) and strength values (yield ~225 Mpa, ultimate ~330 Mpa) in Table 2 were obtained from our tensile tests on the composite to ensure the simulation used accurate material properties.

**Table 3:** Central composite design arrangement and response values at a strain rate of 0.01 s^−1^ [33].

Experiment Number	*f*_0_	*f_n_*	*fc*	*f* *_f_*	*R*_1_	*R*_2_	*R*_3_	*R*_4_
1	0.0001	0.08	0.20	0.90	3.718	844.700	5.163	1689.200
2	0.0001	0.04	0.10	0.85	3.692	799.000	4.900	1661.500
3	0.0002	0.04	0.10	0.85	3.707	799.000	4.336	1760.400
4	0.0001	0.08	0.05	0.88	3.658	890.000	5.544	984.000
5	0.0002	0.04	0.075	0.90	3.715	844.500	3.981	4670.210
6	0.0001	0.08	0.10	0.90	3.818	799.300	4.422	4524.150
7	0.0002	0.06	0.075	0.89	3.094	890.000	4.650	4055.700
8	0.0002	0.04	0.10	0.88	3.428	890.000	3.599	3886.990
9	0.0001	0.08	0.05	0.90	3.758	799.300	5.134	1034.500
10	0.0002	0.06	0.075	0.90	3.328	844.600	4.634	3295.640
11	0.0002	0.04	0.05	0.85	3.188	799.000	3.509	6129.780
12	0.0002	0.06	0.075	0.90	2.983	844.600	4.900	1609.900
13	0.0002	0.08	0.05	0.89	2.988	890.000	4.756	9433.700
14	0.0001	0.06	0.075	0.90	3.448	844.600	4.758	11,099.544
15	0.0001	0.08	0.10	0.90	3.318	890.000	5.131	3373.140
16	0.0001	0.08	0.10	0.90	3.328	890.000	4.268	11,299.895
17	0.0001	0.06	0.075	0.90	3.002	844.600	5.017	3214.460
18	0.0002	0.04	0.10	0.85	3.515	799.000	3.963	2678.190
19	0.0001	0.08	0.05	0.90	3.335	799.300	4.761	7737.760
20	0.0001	0.06	0.10	0.89	3.208	844.600	4.352	6431.840
21	0.0001	0.08	0.05	0.88	3.183	890.000	4.321	2189.140
22	0.0002	0.04	0.05	0.90	3.094	890.000	3.614	10,281.530
23	0.0001	0.04	0.05	0.89	3.185	890.000	5.192	657.400
24	0.0002	0.08	0.05	0.90	3.726	799.300	5.574	77.000
25	0.0001	0.06	0.075	0.88	3.616	799.200	4.398	11,588.561

**Table 4:** The identified GTN parameters at different strain rates.

Strain Rate (s^−1^)	*f*_0_	*f_N_*	*f_c_*	*f_f_*
0.001	0.0048	0.0500	0.0700	0.3200
0.003	0.0046	0.0530	0.0720	0.2800
0.005	0.0044	0.0550	0.0730	0.2500
0.007	0.0043	0.0570	0.0740	0.2200
0.009	0.0042	0.0600	0.0750	0.2000

## Data Availability

The original contributions presented in this study are included in the article. Further inquiries can be directed to the corresponding author.
